# Role of Intravascular Ultrasound in Pulmonary Embolism Patients Undergoing Mechanical Thrombectomy: A Systematic Review

**DOI:** 10.3390/tomography9040111

**Published:** 2023-07-14

**Authors:** Rupak Desai, Maharshi Raval, Kokou Selom Adompreh-Fia, Jai Sivanandan Nagarajan, Nitin Ghadge, Ankit Vyas, Akhil Jain, Timir K. Paul, Rajesh Sachdeva, Gautam Kumar

**Affiliations:** 1 Division of Cardiology, Atlanta VA Medical Center, Decatur, GA 30033, USA; rsachd6@emory.edu (R.S.); gautam.kumar@emory.edu (G.K.); 2 Department of Internal Medicine, Landmark Medical Center, Woonsocket, RI 02895, USA; maharshiraval5897@gmail.com; 3 Department of Internal Medicine, UNT-TCU Medical City Arlington, Arlington, TX 76015, USA; koksel4@gmail.com; 4 Department of Medicine, SRM Institute of Science and Technology, Chennai 603203, India; jaisivanandan@gmail.com; 5 Independent Researcher, Albany, NY 12205, USA; nitinmghadge@gmail.com; 6 Department of Internal Medicine, Baptist Hospitals of Southeast Texas, Beaumont, TX 77701, USA; drankitgvyas@gmail.com; 7 Department of Leukemia, The University of Texas MD Anderson Cancer Center, Houston, TX 77030, USA; akhiljaindr@gmail.com; 8 Division of Cardiology, Saint Thomas Heart Institute, University of Tennessee Health Sciences Center, Nashville, TN 37205, USA; timirpaul@gmail.com; 9 Division of Cardiology, Emory University School of Medicine, Atlanta, GA 30322, USA

**Keywords:** pulmonary embolism, right ventricular function, anticoagulation, thrombolysis, catheter-guided thrombectomy, intravascular ultrasound, contrast

## Abstract

Background: Traditionally, mechanical thrombectomy performed for pulmonary embolism (PE) necessitates the utilization of iodinated contrast. Intravascular ultrasound (IVUS) has been used as a diagnostic and therapeutic modality in the management of acute high and intermediate-risk PE. Recently, with the shortage of contrast supplies and the considerable incidence of contrast-induced acute kidney injury (CI-AKI), other safer and more feasible IVUS methods have become desirable. The purpose of this systematic review was to evaluate the importance of IVUS in patients with PE undergoing thrombectomy. Methods: Medline/PubMed, Embase, Scopus, and Google Scholar were searched for review studies, case reports, and case series. Clinical characteristics, outcomes and the usage of IVUS-guided mechanical thrombectomy during the treatment of acute high and intermediate-risk PE were examined in a descriptive analysis. Results: In this systematic review, we included one prospective study, two case series, and two case reports from July 2019 to May 2023. A total of 39 patients were evaluated; most were female (53.8%). The main presenting symptoms were dyspnea and chest pain (79.5%); three patients (7.9%) presented with syncope, one with shock and one with cardiac arrest. Biomarkers (troponin and BNP) were elevated in 94.6% of patients. Most patients (87.2%) had intermediate-risk PE, and 12.8% had high-risk PE. All patients presented with right-heart strain (RV/LV ratio ≥ 0.9, *n* = 39). Most patients (56.4%) had bilateral PE. Mechanical thrombectomy was performed using IVUS without contrast utilization in 39.4% of the patients. After the initial learning curve, contrast usage decreased gradually over time. There was a significant decrease in the composite mean arterial pressure immediately following IVUS-guided thrombectomy from 35.1 ± 7.2 to 25.2 ± 8.3 mmHg (*p* < 0.001). Post-procedure, there was no reported (0%) CI-AKI, no all-cause mortality, no major bleeding, or other adverse events. There was a significant improvement in symptoms and RV function at the mean follow-up. Conclusions: New evidence suggests that IVUS-guided mechanical thrombectomy is safe, with visualization of the thrombus for optimal intervention, and reduces contrast exposure.

## 1. Introduction

The therapeutic approach for acute intermediate- to high-risk pulmonary embolism (PE) has evolved significantly over the years. With a yearly increase in incidence, PE represents the third leading cause of cardiovascular mortality [1]. PE can present with a variety of clinical signs and symptoms, including shortness of breath, chest pain, hemoptysis, and even syncope or hemodynamic instability. Various biomarkers can be deranged in acute PE, most notably the markers of myocardial injury, like troponin, and the markers of right ventricular (RV) dysfunction, like B-type natriuretic peptide (BNP) or N-terminal (NT)-proBNP [2]. The presence of comorbidities, including hemodynamic instability and right ventricular failure with PE, dramatically affects the prognosis [3]. The European Society of Cardiology (ESC) 2019 guidelines recommend beginning the prognostic assessment and initial risk stratification of suspected or confirmed PE with the presence or absence of hemodynamic instability. In hemodynamically stable patients, further stratification into intermediate- and low-risk categories, with the help of the Pulmonary Embolism Severity Index (PESI) or simplified PESI (sPESI), according to RV dysfunction using echocardiography or computed tomography pulmonary angiography, as well as according to cardiac troponin levels, is recommended with a Class I level of recommendation [2].

For several decades, progressive research has targeted avenues to improve morbidity and mortality from this disease. Recently, new technologies to treat acute PE have emerged [4,5]. Traditionally, the treatment for PE is anticoagulation. However, recently, catheter-guided endovascular therapies for acute PE have gained popularity because of the limitations of both anticoagulation and systemic thrombolysis, the risk of bleeding, and the overall patient condition [6,7]. Compared to systemic thrombolysis, catheter-guided procedures are more effective and safer due to the reduction in the rate of major adverse bleeding events [8]. These techniques rely heavily on pulmonary angiography for procedural guidance. Unfortunately, angiography has limitations due to its poor resolution and inadequate visualization of the vessel lumen, which ultimately affect the accurate identification of thrombi. In addition, the contrast used during pulmonary angiography can increase the risk of contrast-induced acute kidney injury (CI-AKI) and present a challenge in patients with baseline chronic kidney disease (CKD).

In the early 1990s, initial studies using IVUS demonstrated its feasibility and showed promising results in identifying acute PE [9,10]. Evidence has shown that IVUS is more sensitive to the pulmonary arteries when detecting thrombi than angiography, but the clinical impact of these findings has not been elucidated [11,12]. The utilization of IVUS for mechanical thrombectomy in acute PE has not been well documented. Only a single-center prospective study and a few case reports and case series have been reported in the literature. Due to the limited prior available information, alternative treatments, such as IVUS-guided mechanical thrombectomy, are required as a result of the growing concerns surrounding CI-AKI and the limitation of contrast supplies. The objective was to assess the clinical characteristics, usage, and outcomes of IVUS-guided mechanical thrombectomy in the treatment of PE. Here, we present the first systematic review article on the role of IVUS in pulmonary embolism patients undergoing mechanical thrombectomy.

## 2. Materials and Methods

### 2.1. Search Strategy

**Figure 1:**
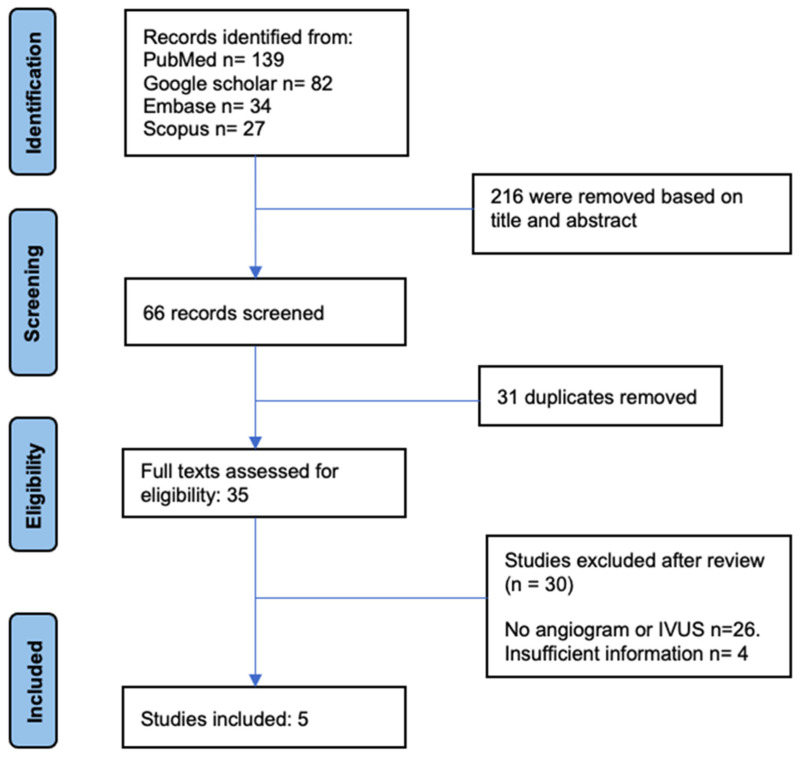
PRISMA diagram for the study population.

We performed a systematic search of the literature, utilizing PubMed, Embase, Scopus, and Google Scholar as our electronic databases. The search methodology was consistent with the PRISMA (Preferred Reporting Items for Systematic Reviews and Meta-Analyses) guidelines. The search was limited to English-language research published between January 2010 and May 2023. The search technique incorporated Medical Subject Headings (MeSH) terminology and keywords associated with intravenous ultrasound, thrombectomy, pulmonary embolism and case reports (Figure 1).

### 2.2. Study Population Selection

We included all published clinical articles investigating IVUS-guided mechanical thrombectomy in acute PE patients. We eliminated studies that lacked sufficient information and did not fit the diagnostic criteria for high- or intermediate-risk PE. No limitation was applied to the methods used to evaluate mechanical thrombectomy. Two reviewers [K.S.A. and N.G.] independently extracted and screened the titles and abstracts of the identified publications, and the full-text articles were retrieved for additional examination. Consensus or consultation with a third reviewer was used to reach agreement [R.D.].

### 2.3. Extraction of Data and Quality Evaluation

The included studies had their data extracted using a standardized data extraction form. Included in the extracted data were the following: (1) clinical presentation of PE (symptoms, laboratory findings, risk stratification, CT findings, ECHO findings); (2) diagnostic criteria for PE; (3) management strategies (imaging, medications, procedures); and (4) outcomes (mortality, complications, recovery). Using the Joanna Briggs Institute critical appraisal technique for case reports, the quality of the included studies was evaluated.

### 2.4. Synthesis and Data Analysis

Given the expected heterogeneity of the included reports and study designs, it was not viable to perform a meta-analysis. Hence, we decided to undertake a narrative synthesis of the data, which included a systematic review and a summary of the findings. The data are provided in tabular and graphical formats. A descriptive analysis was conducted to examine the clinical characteristics, management, and results of IVUS-guided mechanical thrombectomy in the treatment of PE. sPESI was calculated by giving one point each to age > 80 years, history of cancer, history of chronic pulmonary disease, pulse rate ≥ 110 beats per minute, systolic blood pressure < 100 mmHg, and arterial oxyhemoglobin saturation < 90%. All descriptive analyses were performed in Microsoft Excel. Categorical data were represented in numbers or percentages, whereas continuous data were reported as mean ± SD or median with an interquartile range.

## 3. Results

The comprehensive literature review identified 281 studies. Of these, 216 were excluded during the initial screening based on their title and abstract. Of the 65 remaining, 31 duplicates were removed. A total of 35 studies were fully assessed for eligibility. Subsequently, 30 studies still needed to meet the inclusion criteria after the second verification stage. In the end, five studies were included in the systematic review (Figure 1). The included studies consisted of 39 patients (females, 53.84%) who presented with PE and underwent mechanical thrombectomy with IVUS guidance. We included two case reports, two case series, and one prospective study, all from the United States [13,14,15,16,17,18]. Dyspnea and chest pain (79.48%) were the most common presenting symptoms, followed by respiratory failure (10.25%), syncope (7.92%) and cardiac arrest (2.56%). Biomarkers (Troponin and BNP) were elevated in 94.6% of patients (Table 1). Most patients (87.18%) had intermediate-risk PE, and 12.82% had high-risk PE. The most common comorbidities were renal failure/CKD, obesity, and malignancy. Of the 39 patients, a PESI score was reported in 12 patients, and 9 out of 12 had a PESI score between 86 and 188 (75%). A sPESI score was used in the remaining 27 cases, and the sPESI was calculated to be greater than one in 22 out of 27 cases, representing 81.5% of the study population. All patients had evidence of right-heart strain (RV/LV ≥ 0.9). Most patients (56.4%) had bilateral PE.

Mechanical thrombectomy was performed on all 39 patients. In all patients, the Inari FlowTriever^®^ (Inari Medical Inc., Irvine, CA, USA) system was used for mechanical thrombectomy. IVUS alone, without any angiographic contrast agents, was used in 39.4% of patients. In patients in whom contrast agents were used, IVUS guidance decreased the usage of contrast by confirming the exact clot size and localization of the suction catheter. In one case, Ref. [13], simultaneous real-time IVUS was utilized during mechanical thrombectomy via separate venous access.

The composite mean pulmonary arterial pressure decreased significantly immediately following IVUS-guided mechanical thrombectomy, from 35.1 ± 7.2 mmHg to 25.2 ± 8.3 mmHg (*p* < 0.001). In total, 81.81% of patients saw an improvement in dyspnea 48 h after the procedure. RV function improved after the procedure in 92.30% of patients. There was no reported CI-AKI, major bleeding, all-cause mortality, or any other adverse effect following the procedure. All patients survived the event and reported symptom resolution or improvement. For the prospective study [16], all patients (*n* = 26) reported an improvement in dyspnea at a 6-month follow-up. (Table 1 and Table 2).

## 4. Discussion

We conducted a systematic review to identify and analyze the role of IVUS in patients with PE undergoing mechanical thrombectomy. We included five studies with a total of 39 patients, and the findings include a significantly decreased mean pulmonary artery pressure after IVUS-guided mechanical thrombectomy, an improvement in dyspnea, and RV function, with no reported adverse events or mortality.

### 4.1. Epidemiology of Acute PE and Thrombectomy

The prevalence of venous thromboembolic illness (VTE), which can appear as deep-vein thrombosis (DVT), PE, or both, is believed to be between 1 and 2 cases per 1000 people each year. It is the leading preventable cause of death among hospitalized patients in the United States, causing over 100,000 fatalities annually. A sizable portion of acute DVT or PE survivors are at risk of developing debilitating complications such as post-thrombotic syndrome (PTS), recurrent VTE, or chronic thromboembolic pulmonary hypertension, despite receiving anticoagulant therapy (CTEPH) [19].

PE severity is divided into three categories: high-risk (PE with hemodynamic compromise), intermediate-risk PE (PE that results in right ventricular dysfunction that can be seen on an echocardiogram, using computed tomography, or by increased cardiac biomarkers), and low-risk PE (PE without evidence of RV dysfunction or hemodynamic compromise). According to the International Cooperative Pulmonary Embolism Registry (ICOPER), patients with high-risk PE have 90-day death rates of 58.3 percent compared to 15.1 percent for intermediate-risk PE. Studies show that patients with low-risk PE have short-term fatality rates of less than 2% [20].

In recent years, there has been a rise in interest in catheter-based mechanical thrombectomy because of its reduced bleeding risk compared to thrombolytics [21]. Iodinated contrast is used during mechanical thrombectomy [17]. Contrast angiography has a low spatial resolution and insufficient vascular lumen visibility [17]. Additionally, it might be problematic for individuals who already have chronic kidney disease because of the risk of CI-AKI. The uncontrolled studies of CI-AKI after intravenous contrast have noted that the incidence of AKI could be as high as 21% in patients without baseline renal failure and 42% in patients with moderate to severe chronic kidney disease [22]. More controlled studies with propensity score matching note that, although the difference in the development of CI-AKI may not be significant in patients without chronic kidney disease, patients with serum creatinine >2.0 mg/dL have a higher risk of AKI when they receive intravenous contrast compared to when they do not (43.3% vs. 22.5%) [23]. Patients with an estimated glomerular filtration rate of <30 mL/min/1.73 m^2^ are also at a higher risk of AKI with intravenous contrast compared to no contrast (36.4% vs. 19.4%) [24]. Thus, even though the risk of CI-AKI may be lower with the use of intravenous contrast, as opposed to intraarterial contrast, the risk is still significant.

Reduced contrast administration is possible with digital subtraction angiography; however, this may not be possible for most individuals because they cannot hold their breath in the acute PE setting. It has been demonstrated that it is possible to examine the pulmonary artery using intravascular ultrasonography (IVUS), a catheter-based ultrasound imaging method that offers good spatial and temporal resolution without the use of contrast. Early research on acute pulmonary embolism produced outstanding thrombus identification results. It eliminates the requirement for procedural contrast administration when IVUS is used as the only intra-procedural imaging modality in the catheter-directed treatment of PE, which may be especially crucial in patients with pre-existing CKD. Between 5 and 25% of patients presenting with acute PE have been documented to have AKI. The risk of CI-AKI may also be significantly increased using a second contrast load during a endovascular therapeutic procedure after undergoing an initial diagnostic computed tomographic pulmonary angiography (CTPA) [17,25,26].

### 4.2. Overall Impression of Its Efficacy and Impact on Outcomes

Most available data are from a recent study comprising 26 FLASH registry patients who received IVUS-guided thrombectomy between July 2019 and December 2021. Many individuals had high-risk or intermediate-risk PE. The mean baseline composite RV/LV ratio was 1.36 ± 0.27. The volume of contrast agent used decreased over time, and numerous later procedures were completed solely under IVUS guidance. Following thrombectomy, the mean PA pressure significantly decreased from 34.8 ± 8.3 to 25.5 ± 7.3 mmHg (*p* < 0.0001). Additionally, the systolic PA pressure significantly dropped from 55.4 ± 13.9 to 39.5 ± 12.5 mmHg immediately (*p* < 0.0001) [17].

Dyspnea symptoms were evaluated using a modified Medical Research Council (mMRC) score ranging from 0 to 4, which had improved from 2.6 at baseline to 0.9 at 48 h and to 0.5 at 6 months. Severe dyspnea, as noted by an mMRC score of 3 or 4, reduced significantly from 68% at baseline to 5.6% at 48 h and 5.3% at 6 months [17]. Using echocardiography at baseline and at 6 months, the RV function was evaluated. However, as not all patients had completed a 6-month follow-up, the RV function was reported from the latest follow-up echocardiogram. Compared to the baseline, there were significant improvements in the echocardiographic evaluations of the RV function at a mean follow-up of 87 days, with the RV/LV ratio improving from 1.19 to 0.86, the proportion of patients with normal or mildly reduced RV function increasing from 26.3% to 100%, and the proportion of patients with a normal RV size increasing from 15.8% to 84.6% [17].

The use of IVUS to confirm thrombus aspiration following catheter-directed mechanical thrombectomy was documented in an initial case series [14]. However, the use of IVUS to direct the aspiration was not performed in real-time in those cases due to the requirement for additional venous access, which may have contributed to increased complications. There has been one case report of real-time simultaneous IVUS use during catheter-directed mechanical thrombectomy without the need for additional angiograms until now [27,28]. A comprehensive embolectomy might not be required to enhance RV function. The presence of a remnant thrombus, however, can have medium- and long-term adverse effects, such as a diminished functional ability or persistent thromboembolic pulmonary hypertension. Utilizing IVUS reduced the exposure to contrast and the required number of catheter changes, while facilitating the accurate localization of the suction catheter and the sizing of the embolectomy disks [13,14,15,16,18,26,28]. Reduced contrast exposure is associated with a reduced risk of CI-AKI, which is associated with worse short and long-term outcomes. A retrospective case-matched cohort study by From et al. noted that CI-AKI is associated with a higher 30-day mortality and overall mortality after the adjustment of various comorbidities, with odds ratios of 3.37 (*p* < 0.001) and 1.57 (*p* < 0.001), respectively. The use of intravenous contrast was also noted to be a risk factor for 30-day and overall mortality, with odds ratios of 2.91 (*p* = 0.02) and 3.01 (*p* < 0.001), respectively [29]. A prospective study of 633 patients also noted that CI-AKI was associated with higher numbers of 1-year major adverse events, with an adjusted incident risk ratio of 2.36 [30]. Thus, minimizing the risk of CI-AKI may improve the outcomes of these patients undergoing IVUS-guided mechanical thrombectomy.

### 4.3. Cost-Effectiveness

Most of the data regarding the cost-effectiveness of IVUS are from patients undergoing percutaneous coronary intervention (PCI). Less than 10% of PCI procedures in the US and far fewer in Europe and Australia employ IVUS [27]. One of the main obstacles to IVUS use, according to a recent study of interventional cardiologists in practice, was the procedure’s alleged high cost. This could account for why IVUS is used more frequently in nations like Japan, where the operation is paid for separately.

A health economic analysis was recently conducted in Australia comparing IVUS with angiographically guided drug-eluting stents. The cost-effective subgroup analysis of this study showed that the additional cost of using an IVUS-guided catheter was approximately USD 1241. Secondly, it was also noted that the incremental cost-effectiveness of IVUS-guided angiography compared to conventional angiography was approximately USD 5210 per quality-adjusted life year. Similarly, another study conducted in Italy showed similar cost-effectiveness when using IVUS-guided angiography compared to conventional angiography [28,31].

Given the limited data, such a cost-effective analysis of IVUS-guided mechanical thrombectomy for PE is not available. IVUS is mostly used as an imaging support in PE, unlike in PCI, where it is used to determine the need for the revascularization and placement of drug-eluting stents. Despite this, the use of IVUS is expected to be more cost-effective in mechanical thrombectomy, given its accuracy in detecting thrombus [11], ability to reduce the procedural time and reduce periprocedural complications like AKI by reducing the use of contrast, thereby reducing the length of stay, and improving patient outcomes.

### 4.4. Catheter-Based Mechanical Thrombectomy and Surgical Pulmonary Embolectomy

Several techniques and catheters have been used for this purpose. Only the more promising or commonly used techniques are discussed here. Three main catheters used for mechanical thrombectomy include Inari (Inari Medical^®^, Irvine, CA, USA), Penumbra (Pneumbra Inc., Alameda, CA, USA), and Angiovac (Angiodynamics, Latham, NY, USA). Although a preprocedural computed tomography angiogram is often the only guidance needed for the placement of catheter-directed lysis catheters, selective pulmonary angiograms are typically used in mechanical thrombectomy cases to assess the location of emboli, potential targets for treatment, the choice of thrombectomy device, and the best projection during angiography to optimize catheter navigation [32].

Before 2008, mechanical fragmentation performed by a pigtail catheter was used in 70% of catheter-based therapies [20]. However, ultrasound-associated thrombolysis surpassed mechanical fragmentation as the primary catheter-based therapy after the FDA approval of EKOS^®^ (Boston Scientific, Marlborough, MA, USA). Aspiration therapy, capable of more rapid thrombus elimination under the guidance of IVUS, may offer a viable therapeutic alternative because catheter-directed lysis sometimes necessitates more than 12 h of therapy. A few studies are available to support the use of these newer techniques. However, multiple studies are currently ongoing in the field of PE management.

The EXTRACT-PE (Evaluating the Safety and Efficacy of the Indigo Aspiration System in Acute Pulmonary Embolism) study of the Indigo Aspiration device for intermediate-risk PE found a statistically significant reduction in the RV/LV ratio (0.43; 95% CI: 0.38 to 0.47; *p* < 0.0001), an average reduction in the systolic PA pressure of 7.9%, and a low major adverse event rate (1.7%) following use of the device, with 98.3% of patients not receiving any pre-procedure tPA. There were no hemorrhagic strokes, and the rate of severe bleeding was 1.7%. The EXTRACT-PE study’s RV/LV ratio reduction of 0.43 at 48 h was similar to the results of earlier catheter-directed treatment studies [33]. To our knowledge, there are no reports published in the literature indicating that IVUS has been used before or after penumbra mechanical thrombectomy. Patients using the Inari FlowTriever System saw an average reduction in the RV/LV ratio of 0.38 in the FLARE (a prospective, single-arm, multicenter trial of catheter-directed mechanical thrombectomy for intermediate-risk acute pulmonary embolism) research, which prospectively examined the device [4,14,34].

Multiple larger RCTs comparing catheter-directed thrombolysis (CDT) plus Anticoagulation versus systemic anticoagulation alone in patients with intermediate-risk PE are ongoing, of which the results of the HI-PEITHO (ultrasound-facilitated, catheter-directed, thrombolysis in intermediate–high-risk pulmonary embolism, NCT04790370) trial is the most awaited of all trials. The PEERLESS trial (which compares the FlowTriever System to CDT for treating acute pulmonary embolism, NCT05111613) is another interesting ongoing trial. Finally, the Angiovac system comprises an Angiovac circuit, an extracorporeal bypass tubing system, and an Angiovac cannula, intended for venous drainage during extracorporeal bypass, which has been used in some case reports in patients with intermediate- and high-risk PE. However, collecting clinical data from RCTs is still an area of active investigation [32,35,36,37,38].

Surgical pulmonary embolectomy (SPE), which is traditionally used in patients with hemodynamically unstable PE who have experienced absolute contraindications to thrombolysis, failed systemic thrombolysis or catheter-based treatment, or in patients in whom shock would result in death before systemic thrombolysis could take effect, has now been noted to be a safe and viable option for patients with submassive and massive PE, irrespective of hemodynamic instability [39]. In a retrospective study of 105 patients that underwent SPE for acute massive or submassive PE, acceptable operative and 1-year mortality rates were noted at 6.7% and 20.0%, respectively [39]. Another retrospective study of 55 patients noted its excellent safety, with in-hospital and 1-year survival at 93% and 91%, respectively [40]. Despite being a more invasive modality, SPE may be more cost-effective compared to catheter-based mechanical thrombectomy, and given its safety, large-scale randomized studies are recommended to determine whether SPE can be offered as a first-line treatment option for patients with massive and submassive PE.

### 4.5. Strengths and Limitations of IVUS

IVUS has been mentioned as a treatment option for people with acute PE. The following are the benefits of IVUS use in patients: (1) greater diagnostic accuracy due to improved artery visibility and thrombus detection over angiography; (2) greater procedural success and fewer problems in individuals with subacute and acute PE; (3) a reduced exposure to contrast and fluoroscopy resulting in better outcomes for CKD patients; (4) assistance in the identification of anatomical details such as the vessel lumen size and of thrombi characteristics, such as location, size, mobility, and chronicity, and assistance in deciding upon management choices, such as improved precision regarding the placement of the aspiration catheter and decreasing the frequency of blind aspirations, ultimately reducing blood loss.

Some limitations are worth mentioning regarding the utility of IVUS during mechanical thrombectomy. The length of the 0.035″ IVUS catheter is insufficient for the adequate visualization of the pulmonary tree through the Triever catheter. For a complete assessment of the pulmonary tree, the IVUS catheter must be passed initially, followed by the Triever catheter, which may increase the procedure time, as was shown in the study [16]. To overcome this limitation, one can use a 0.018″ IVUS catheter. However, the image field becomes restricted because of the small field of view and large size of these vessels.

### 4.6. Strengths and Limitations of This Review

This is a comprehensive review of all the case studies and research articles available on the role of IVUS in PE and mechanical thrombectomy. We discuss various aspects of this technique, including its efficacy, impact on outcomes, cost-effectiveness, alternative options, and finally, the usefulness and limitations of IVUS. In this regard, we offer comprehensive knowledge that can be obtained from a single source and cover all relevant facts. A significant limitation of this review is the need for more literature in this area to provide conclusive evidence. The research is preliminary, and there is a need for further investigation in the field. Data are available for only one IVUS catheter, and the catheter resolution and image quality may have had an impact on the outcomes of interest. The information obtained is mainly from case reports and one study, while other information needs to be extrapolated from the data available for different indications of IVUS. Given that we explore the potential of IVUS at times by drawing from related fields, the scientific gain may be viewed as small at this stage.

### 4.7. Future Directions

The therapies for acute PE recommended by the guidelines include anticoagulation or systemic thrombolytics in cases of hemodynamic compromise, with catheter-based therapy, including mechanical thrombectomy, being reserved for cases in which systemic thrombolytics have failed to achieve the desired results or are contraindicated. The case for mechanical thrombectomy as the first-line treatment for PE is becoming more and more compelling. Yet, more information on IVUS-guided mechanical thrombectomy procedures has to be published. In acute PE, it is possible to use IVUS-guided mechanical thrombectomy with little or no angiographic contrast. This is a safe and viable alternative to mechanical thrombectomy with contrast angiography, and is particularly important for individuals with CKD or other risk factors for CI-AKI, as well as in scenarios of contrast agent shortage. As technology advances and research continues to unfold, there are several potential avenues for further exploration. Firstly, there is a need for more extensive, multicenter studies to assess the long-term outcomes and safety profile of IVUS-guided mechanical thrombectomy in PE patients. These studies would provide more robust evidence and further establish the efficacy of this approach. Furthermore, ongoing research efforts should focus on optimizing the technical aspects of IVUS, such as improving catheter designs, enhancing image quality, and refining the techniques used for accurate clot visualization and removal. Ultimately, the future of IVUS in PE management lies in its continuous integration with advanced technologies and comprehensive evaluation through rigorous clinical trials, with the ultimate goal of improving patient outcomes and revolutionizing the field of PE treatment.

## 5. Conclusions

The use of IVUS for mechanical thrombectomy in acute PE is technically feasible and has a favorable safety profile. This could be particularly important for individuals with CKD or other risk factors for CI-AKI. As new technology emerges and expertise advances, IVUS will likely become a prominent tool in the management of PE. Nonetheless, further randomized controlled trials and comparative data analyses are required.

## Figures and Tables

**Table 1:** Patient Characteristics.

Characteristics	*n* (%) or Mean ± SD	N
Age	55 ± 15	39
Sex, Female	21 (53.84%)	39
Presenting symptoms		
Dyspnea + Chest pain	31 (79.48%)	39
Syncope	3 (7.92%)	39
Respiratory failure	4 (10.25%)	39
Cardiac arrest/PEA	1 (2.56%)	39
Type of PE		
Saddle	12 (30.77%)	39
Unilateral	3 (7.69%)	39
Bilateral	22 (56.41%)	39
Saddle + RPA + LPA	28 (71.79%)	39
Elevated biomarkers		
Trop, BNP	35 (94.59%)	37

Abbreviations: PE = Pulmonary Embolism, Trop = Troponin, BNP = Brain Natriuretic Peptide, RPA = Right Pulmonary Artery, LPA = Left Pulmonary Artery, PESI = Pulmonary Embolism Severity Index, sPESI = Simplified Pulmonary Embolism Severity Index.

**Table 2:** Role of Intravascular Ultrasound System (IVUS) in PE patients undergoing mechanical thrombectomy.

Reference (Author, Year, Country)	Age, Sex	Presenting Complaints	H/O Comorbities	RV/LV Ratio	Type of PE	PESI/SPESI Score	System Used for Thrombectomy	Mean PAP (in mmHg) (before ⇒ after)	Any Additional Intervention	Outcome
Case reports										
Bertot et al., 2023, USA [16]	80, F	Syncope	Renal failure	>0.9	Bilateral	-	Catheter thrombectomy using IVUS only		Right ventricular assistance device used	Resolution of shock and RHF
Hassanin et al., 2022, USA [14]	46, M	Dyspnea, chest pain, near syncope	Liver transplantation, Renal failure, Heart failure	1.8	RPA + LPA	sPESI = 1	Catheter-directed embolectomy (FlowTriever, Inari Medical) with simultaneous IVUS guidance	48 ⇒ 19	Nil	Dyspnea resolved
Case series										
Kumar et al., 2022, USA [15]	30, F	Dyspnea, chest pain	Obesity, H/O Left pelvis and toe fractures	1.33	Saddle + RPA + LPA	PESI = 50	Percutaneous pulmonary embolectomy using the INARI FlowTriever system	34 ⇒ 25	Nil	Significant improvement in dyspnea
Kumar et al., 2022, USA [15]	53, M	Hypoxic respiratory failure	Obesity BMI 42, CAD, Multiple rib fractures × 2 days	0.99	Saddle + RPA + LPA	PESI = 83	IVUS-guided only catheter-directed aspiration thrombectomy	31 ⇒ 25	Mechanical ventilation	Improvement in O2 sat
Kumar et al., 2022, USA [15]	61, F	Exertional dyspnea	CKD, H/O PE, Obesity (BMI 47)	2.05	Saddle + RPA + LPA	PESI = 101	Percutaneous pulmonary thrombo-embolectomy under IVUS guidance only	53 ⇒ 49	Nil	Dyspnea and oxygen saturation improved
Kumar et al., 2022, USA [15]	67, F	Syncope	CKD, Colon CA	1.86	Saddle + RPA + LPA	PESI = 97	IVUS-guided aspiration thrombectomy using the INARI FlowTriever system	31 ⇒ 26	Nil	
Kumar et al., 2022, USA [15]	68, F	Dysnea, chest pain	CKD, CAD, DM, recent GI bleed, Obesity	2.5	Saddle + RPA + LPA	PESI = 88	IVUS-guided aspiration thrombectomy using the INARI FlowTriever system	24 ⇒ 18	Nil	
Kumar et al., 2023, USA [15]	30, F	SOB	H/O MVC × 12 days back, conservative management	1.47	Saddle	PESI = 110	Mechanical thrombectomy using INARI FlowTriever system	34 ⇒ 25	Nil	Complete resolution of symptoms
Kumar et al., 2023, USA [15]	68, M	PEA on POD-1	H/O MVC–liver laceration, small bowel injury requiring exploratory laparotomy, small bowel resection, HTN, T2DM	1.32	Saddle	PESI = 188	Mechanical thrombectomy using INARI FlowTriever system	43 ⇒ 36	Mechanical ventilation	Recovered
Kumar et al.,2023,USA [15]	44, M	Hypoxemia on day 4 of hospitalization	Ground-level fall due to spinal canal stenosis	1.9	Bilateral	PESI = 124	Mechanical thrombectomy using INARI FlowTriever system	62 ⇒ 54	Mechanical ventilation	Recovered, Expired after 5 months due to cardiac arrest caused by recent/remote PE
Kumar et al., 2023, USA [15]	53, M	Acute hypoxic respiratory failure on POD-1	MVC causing multiple rib fractures and flail chest requiring surgical intervention, HTN, HFrEF	0.92	Saddle	PESI = 113	Mechanical thrombectomy using INARI FlowTriever system	31 ⇒ 25	Mechanical ventilation	Recovered
Kumar et al., 2023, USA [15]	77, M	Syncope and fall	Ground-level fall c/b nasal bone fracture and soft tissue laceration of face, HTN, T2DM, H/O CVA	1.43	Saddle	PESI = 97	Mechanical thrombectomy using INARI FlowTriever system	36 ⇒ 23	None	Recovered
Kumar et al., 2023, USA [15]	51, F	Acute onset hypoxic respiratory failure on POD-3	MVC c/b open tibial fracture requiring urgent surgery, HTN	1.8	Bilateral	PESI = 91	Mechanical thrombectomy using INARI FlowTriever system	36 ⇒ 26	Three pressors	Expired on hospital day 16 due to worsening ARDS, septic shock c/b multi organ failure
Prospective										
Kumar et al., 2023, USA (*n* = 26) [17]	Mean age 54.7 ± 13.5 Sex, F = (53.8%)	Dyspnea			Saddle = 8 (30.8%) Unilateral = 3 (11.5%) Bilateral = 15 (57.7%)	sPESI 1.9 ± 1.2	IVUS-guided mechanical thrombectomy using INARI FlowTriever system	34.8 ± 8.3 ⇒ 25.5 ± 7.3	Nil	Improvement in dyspnea (nMRC score) Baseline = 2.6 ± 1.4 48 h = 0.9 ± 1.2 6 months = 0.5 ± 0.8

Abbreviations: PE = Pulmonary Embolism. CAD = Coronary Artery Disease, CA = Cancer, MVC = Motor Vehicle Collision, c/b = complicated by, PESI = Pulmonary Embolism Severity Index, sPESI = Simplified Pulmonary Embolism Severity Index, PAP = Pulmonary Artery Pressure, RHF = Right Heart Failure, ARDS = Acute Respiratory Distress Syndrome.

## Data Availability

No new data were created or analyzed in this study. Data sharing is not applicable to this article.
